# Dual Effects of Alpha-Arbutin on Monophenolase and Diphenolase Activities of Mushroom Tyrosinase

**DOI:** 10.1371/journal.pone.0109398

**Published:** 2014-10-10

**Authors:** Liang Qin, Yang Wu, Youting Liu, Yiming Chen, Peng Zhang

**Affiliations:** Beijing Bioprocess Key Laboratory, College of Life Science and Technology, Beijing University of Chemical Technology, Beijing, China; Institute of Plant Physiology and Ecology, China

## Abstract

The effects of α-arbutin on the monophenolase and diphenolase activities of mushroom tyrosinase were investigated. The results showed that α-arbutin inhibited monophenolase activity but it activated diphenolase activity. For monophenolase activity, IC_50_ value was 4.5 mmol·L^−1^ and 4.18 mmol·L^−1^ of α-arbutin could extend the lag time from 40.5 s to 167.3 s. Alpha- arbutin is proposed to be regarded as a triphenolic substrate by the enzyme during catalyzation, leading to the suicide inactivation of the active site of tyrosinase. For diphenolase activity, α-arbutin acted as an activator and its activation mechanism was mixed type activation. To reveal such activation, it should be mainly refered to the conformational changes in tyrosinase caused by the interaction of α-arbutin with residues located at the entrance to the active site, and the decrease of the effect of suicide inactivation.

## Introduction

Tyrosinase (EC 1.14.18.1), also called polyphenol oxidase [1], is a copper containing mixed-function enzyme. It is widely distributed in animals, plants, fungi and microorganisms. Enzymatic browning in vegetables and fruits and the formation of the melanin of skin, hair, and eye are caused by the activity of the tyrosinase. Tyrosinase catalyzes two different reactions in the presence of molecular oxygen: the hydroxylation of monophenol (monophenolase activity) and the oxidation of o-diphenol to o-quinone (diphenolase activity) [2]–[3]. The crystallographic structure of tyrosinase has been reported, and the active site of tyrosinase is composed of six conserved histidine residues which coordinate two copper ions, denoted Cu_A_ and Cu_B_ [1], [4]. Tyrosinase is the key enzyme during the formation of melanin and melanin is effective in preventing skin injury by UV and it plays a major role in formation of skin color [5]–[6]. However, pigmentation disorders (melasma, freckles, senile lentigines, etc.) will cause a serious esthetic problem in human beings and these symptoms become eminent with aging [7]. It was reported that mushroom tyrosinase can be inhibited by aromatic aldehydes [8], aromatic acids [9], tropolone [10] and kojic acid [11] and quercetin [12] and so on. Tyrosinase inhibitors have become increasingly important in medicinal [13] and cosmetic [14] products. Inhibition on the tyrosinase activity will contribute to the treatment of the pigmentation of skin diseases and the prevention of enzymatic browning of vegetables. Tyrosinase has great potential for the production of various o-diphenols. Diphenols as intermediates play important role in the synthesis of pharmaceuticals, agrochemicals, flavors, polymerizationinhibitors, and antioxidants [15]–[18]. However, the application of tyrosinase for catechol synthesis has been restricted since its diphenolase activity is much greater than its monophenolase activity [19].

Arbutin is well known to be added into cosmetics as whitening ingredient and it also has many other functions, such as bactericidal and anti-inflammatory effects. Inhibitory effect of α-arbutin on tyrosinases from mushroom, B16 mouse melanoma and HMV-II human melanoma cells has been investigated previously [20]–[22]. In fact, tyrosinases can also be activated by many substances in crude tissue preparation. It has been reported that the latent tyrosinase from plant and insect sources can be activated by different treatments or agents, such as SDS [23], fatty acids [24], alcohols [25] and pathogen attack [26]. However, no reports about the dual effects of α-arbutin on monophenolase and diphenolase activities of mushroom tyrosinase have been published.

In this work, to obtain more detail information about the effects of α-arbutin on mushroom tyrosinase, monophenolase and diphenolase activities were studied, respectively. Activatory effect of α-arbutin on diphenolase activity is unexpected, but finally we found the breakthrough to explain this phenomenon. We analyzed the dual effects on mushroom tyrosinase by α-arbutin from aspects of suicide inactivation to reveal monophenolase inhibition and conformational changes to illuminate diphenolase activation. The study might guide significance for the design of new drugs in terms of whitening agents and blackening agents, such as skin whitening, hair blackening agent and so forth, and provide another way to the treatment of pigment synthesis disorders.

## Materials and Methods

### 1. Materials

Mushroom tyrosinase (EC 1.14.18.1, 8300 units/mg), Alpha-arbutin were supplied by Sigma. Although mushroom tyrosinase differs somewhat from other sources and it contained several isoforms that most of the enzyme is E_met_ form and a small fraction is present as E_oxy_ [27]–[28], it is used for study because of its ready availability [29]–[30]. Protein concentrations were determined by using Bradford's method [31] using BSA as a standard. The substrates used, L-Tyrosine and L-Dopa, were all from Sigma; Na_2_HPO_4_, NaH_2_PO_4_, were of analytical grade (Beijing Chemical Reagent Company, Beijing, China); DMSO (Dimethyl sulfoxide), hydroquinone, methanol and acetic acid (HPLC grade, Sigma-Aldrich, St. Louis, MO, USA); All other chemicals were of analytical grade and supplied by Merck (Frankfurt, Germany). Cary50 UV-visible spectrophotometer (Varian Co., UK); LC-10ATVP HPLC (Shimadzu Co., Japan).

### 2. Methods

Ethics Statement: N/A.

#### 2.1 Enzyme activity assay

The activities of monophenolase and diphenolase were determined by measuring the rate of dopachrome formation at 475 nm (ξ = 3700 M^−1^·cm^−1^) with Cary50 UV-visible spectrophotometer (Varian Company).The assay was carried out in air-saturated solutions and freshly prepared enzyme and substrate solutions were used in this study. One unit (U) of enzymatic activity was defined as the amount of enzyme increasing 0.001 absorbance·min^−1^ at 475 nm. The reaction was controlled under the temperature of 30°C and pH of 6.8 to stabilize dopachrome. Steady state rate was defined as the slope of the linear range of dopachrome accumulation curve. And the initial reaction velocity (mol·L^−1^·min^−1^) was calculated as follows:
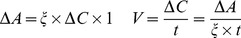
(2.1)


Where, A is absorbance value; *ξ* = 3700 M^−1^·cm^−1^; C is solution concentration (mol·L^−1^); *V* is initial reaction velocity (mol·L^−1^·min^−1^).

### 2.2 The determination of monophenolase activity and diphenolase activity

L-Tyrosine and L-Dopa were used as the substrates to determine the monophenolase activity and diphenolase activity, respectively. In this method, the reaction media (3.0 mL) contained 0.5 mM L-Tyrosine or L-Dopa in 50 mM phosphate buffer (pH 6.8), α-arbutin solution (different concentrations) and mushroom tyrosinase. The final concentration of mushroom tyrosinase was 20.0 µg·mL^−1^ for the monophenolase activity and 6.67 µg·mL^−1^ for the o-diphenolase activity. Firstly, 0.1 mL of different concentrations of α-arbutin dissolved in 3% DMSO (Dimethyl sulfoxide) solution was added into the test tube. Then, 2.8 mL substrate solution in Na_2_HPO_4_–NaH_2_PO_4_ buffer was mixed. In condition of pH 6.8, the mixture, without enzyme of mushroom tyrosinase, was incubated at 30°C for 10 min and the reaction was initiated by the addition of enzyme. The assay was carried out in air-saturated solutions and freshly prepared enzyme and substrate solutions were used in this study. Tyrosinase is known to catalyze a reaction between two substrates, a phenolic compound (L-Dopa) and oxygen. Therefore, the effect of oxygen concentration on these parameters is unknown.

According to the definition of enzyme activity, inhibition rate (*I* %) of tyrosinase activity (monophenlase activity and diphenolase activity) by α-arbutin was calculated as follows.
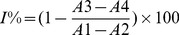
(2.2)


In the formula, *A*_1_ is the system without α-arbutin; A_2_ is the system without α-arbutin and substrate; *A*_3_ is the system including all solutions; *A*_4_ is the system without substrate but including α-arbutin. If the inhibition rate was negative, it meant the tyrosinase was activated. The activation rate (*A* %) was the absolute value of inhibition rate (*I* %).

### 2.3 The determination of kinetic parameters and kinetic type

The inhibition type and kinetic constants were determined by the plots of Lineweaver-Burk and changes of the constants (K_m_, apparent Michaelis constant of tyrosinase towards o-diphenol and V_m_, apparent maximum steady-state rate of tyrosinase towards o-diphenol) of Michaelis-Menten (M-M) equation [32].

## Results

### 1. Inhibitory effect of α-arbutin on monophenolase of mushroom tyrosinase

**Figure 1:**
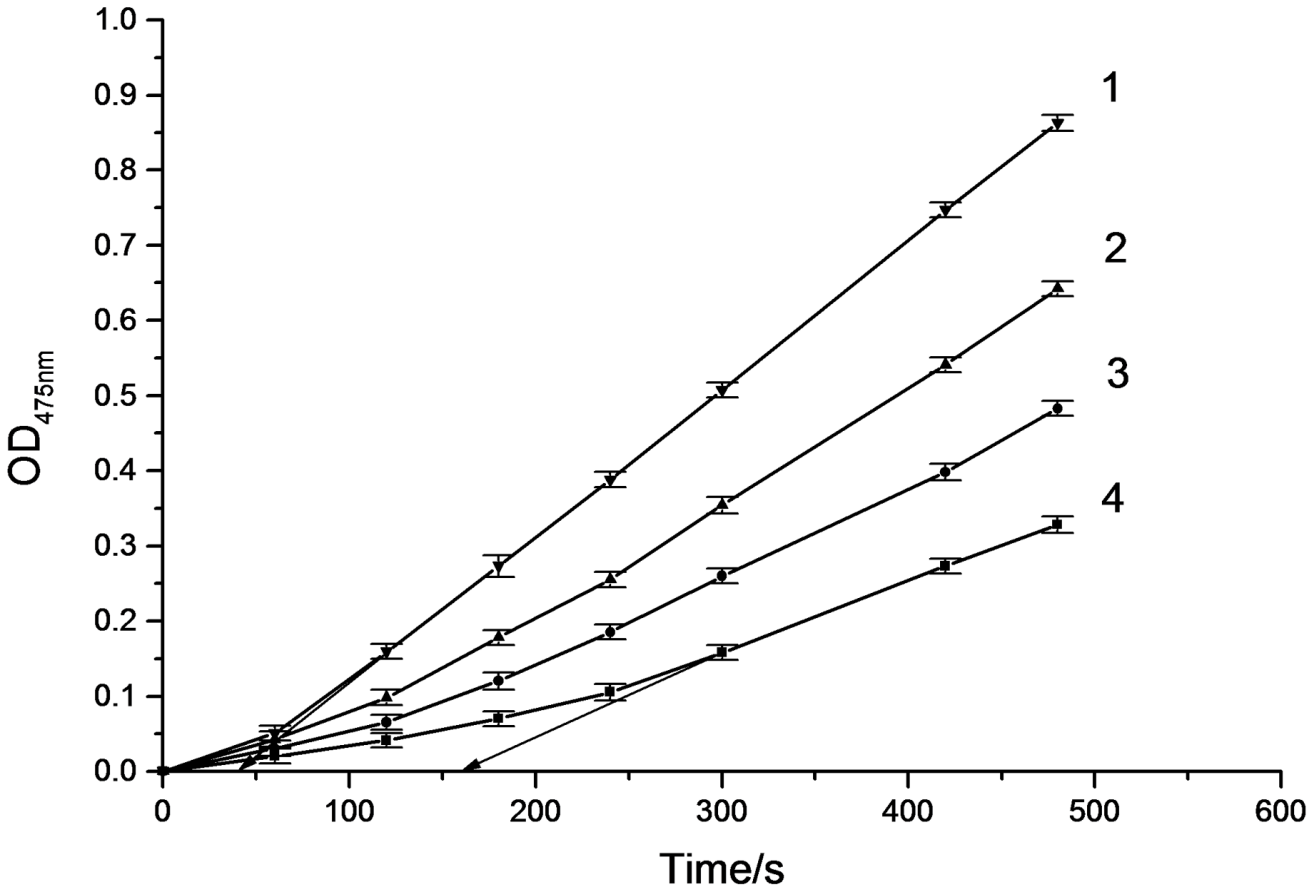
Progress curves for the inhibition of monophenolase of mushroom tyrosinase by α-arbutin at 30°C. The reaction media (3.0 mL) contained 0.5 mM L-tyrosine in 50 mM phosphate buffer (pH 6.8), the indicated concentration of α-arbutin, and mushroom tyrosinase (20 µg/mL). The concentrations of α-arbutin for curves 1∼4 were 0, 1.67, 3.34, 4.18 mmol·L^−1^. The reaction was started by the addition of the enzyme.

**Figure 2:**
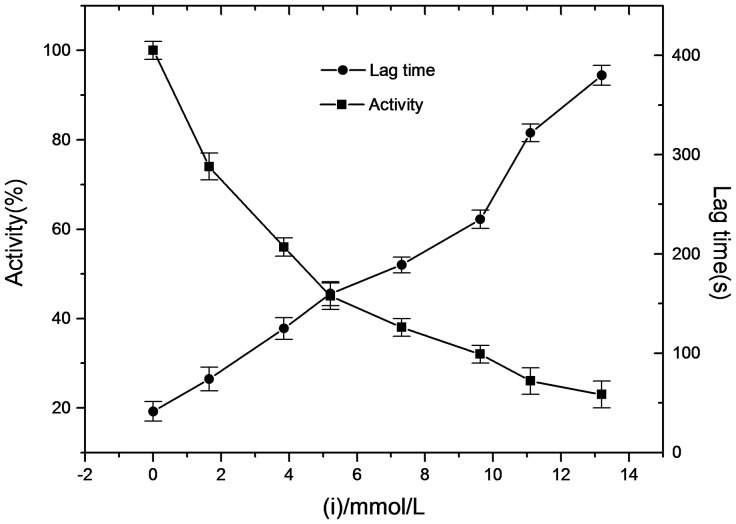
Effects of α-arbutin on the enzyme activity and the lag time of monophenolase activity of mushroom tyrosinase. Assay conditions: 3.0 ml 50 mM phosphate buffer pH 6.8, containing 0.5 mM L-tyrosine. The reaction was started by the addition of the enzyme (20 µg/mL).

The inhibitory effect of α-arbutin on monophenolase of mushroom tyrosinase was investigated, and the reaction process curve of catalyzing L-Tyrosine into dopachrome was shown in Fig. 1. The result showed that the lag time of α-arbutin for monophenolase activity was extended significantly, from 40.5 s to 167.3 s, with 4.18 mmol·L^−1^ α-arbutin. The system reached steady state rate after the lag period. Inhibition of the enzyme by α-arbutin was concentration-dependent as shown in Fig. 2. The enzyme activity, which was the slope of the linear range of the kinetic curve, was reduced with the concentration of α-arbutin increasing in the reaction system. When the concentration of α-arbutin reached to 4.5 mmol·L^−1^, the relative enzyme activity was determined to be 50%, which meant the inhibition rate (IC_50_) of α-arbutin on monophenolase was 4.5 mmol·L^−1^. So the results illustrated that inhibition effect of α-arbutin on monophenolase of mushroom tyrosinase was to extend the lag time and reduce the enzyme activity in the steady state.

### 2. Activatory effect of α-arbutin on diphenolase of mushroom tyrosinase

**Figure 3:**
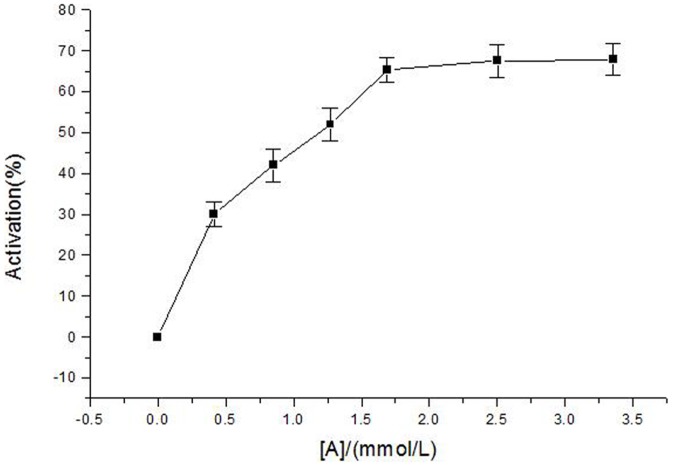
Activation rate of diphenolase of mushroom tyrosinase by α-arbutin. Assay conditions: 3.0 ml 50 mM phosphate buffer pH 6.8, containing 0.5 mM L-Dopa, different concentrations of α-arbutin and mushroom tyrosinase (6.67 µg/mL).

The activity of diphenolase of mushroom tyrosinase used L-Dopa as substrate was investigated. The course of enzyme reaction was shown in Fig. 3. It showed that α-arbutin had activated effect on the diophenolase activity of mushroom tyrosinase. When the catalyzed reaction system contained α-arbutin, the synthesis of dopachrome was promoted. Initially, the activation rate increased by increasing the concentration of α-arbutin, but when the concentration of α-arbutin reachd a certain value (at around 2.5 mmol·L^−1^), the activation rate was steady. That means, α-arbutin plays an activator in the reaction of catalyzing L-Dopa into dopachrome by diphenolase.

### 3. Determination of the kinetic parameters and kinetic type of α-arbutin on diphenolase

**Figure 4:**
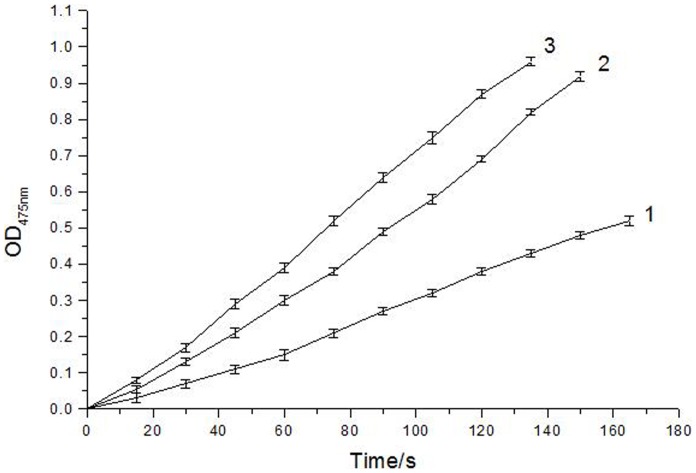
Progress curves for the activation of diphenolase of mushroom tyrosinase by α-arbutin . The reaction media (3.0 mL) contained 0.5 mM L-Dopa in 50 mM phosphate buffer (pH 6.8), the indicated concentration of α-arbutin, and mushroom tyrosinase (6.67 µg/mL). The concentrations of α-arbutin for curves 1∼3 were 0, 5, 10 mmol·L^−1^.

The kinetic of mushroom tyrosinase during the oxidation of L-Dopa was studied. Under the condition employed in the present investigation, the oxidation reaction of L-Dopa by mushroom tyrosinase follows Michaelis-Menten kinetics. In the reaction system, we kept constant final concentrations of enzyme and L-Dopa, changed the concentration of α-arbutin to get the progress curve (Fig. 4). The results showed that, there was no lag time for α-arbutin on diphenolase activity.

**Figure 5:**
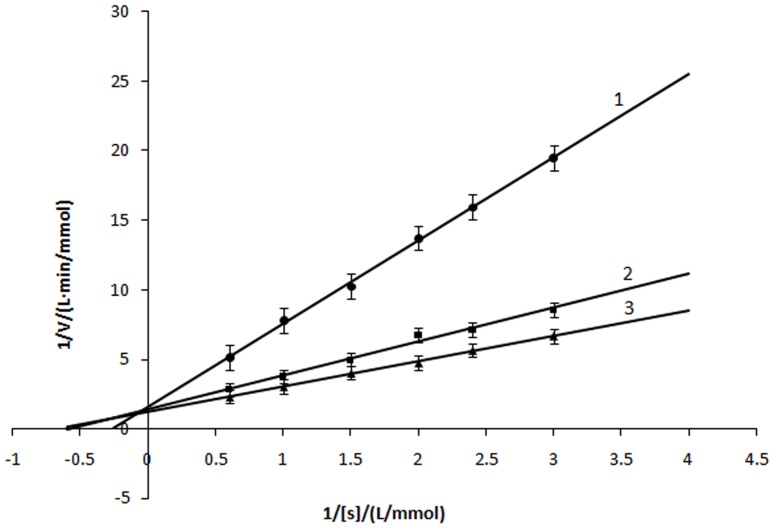
Lineweaver-Burk plots for activation of α-arbutin on mushroom tyrosinase for the catalysis of L-Dopa at 30°C, pH 6.8. The reaction media (3.0 mL) contained 50 mM phosphate buffer (pH 6.8), different concentrations of L-Dopa assubstrate,different concentrations of α-arbutin and mushroom tyrosinase (6.67 µg/mL). Concentrations of α-arbutin for curves 1∼3 were 0, 5, 10 mmol·L^−1^, respectively.

Furthermore, we kept constant final concentration of enzyme, changed the concentration of L-Dopa to measure effects of different concentrations of α-arbutin on diphenolase activity. Kinetic parameters for the diphenolase activity of mushroom tyrosinase were shown in Fig. 5 as Lineweaver-Burk plot.

The initial reaction velocity could be calculated by formula 2.1. And changes of the constants (K_m_ and V_m_) of Michaelis-Menten (M-M) equation could be determined through the plots of Lineweaver-Burk, to determine the kinetic type.

Kinetic parametes of diphenolase by α-arbutin were shown in Table 1, it showed that K_m_ decreased and V_m_ increased as the increasing of α-arbutin concentration, which illustrated that the affinity between diphenolase and α-arbutin. Therefore, we concluded that α-arbutin acted as an activator on diphenolase activity and its activation mechanism was a competitive-uncompetitive mixed activation type.

**Table 1:** Kinetic parametes of diphenolase by α-arbutin.

C_A_(mmol·L^−1^)	L-B equation	K_m_(mmol·L^−1^)	V_m_(mmol·L^−1^min^−1^)
0	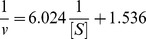	3.922	0.6510
0.76	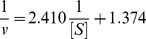	1.754	0.7278
1.52	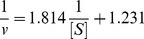	1.474	0.8123

## Discussion

**Figure 6:**
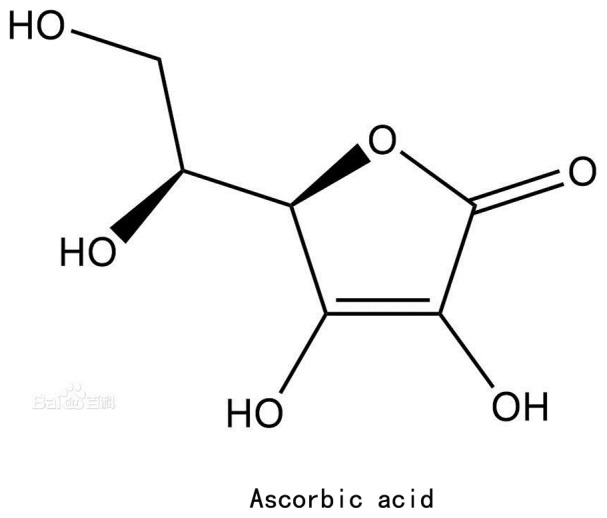
The chemical structure of ascorbic acid.

From the experimental results above,there is an interesting phenomenon that α-arbutin presents dual effects on monophenolase and diphenolase activities of mushroom tyrosinase. For the monophenolase reaction, α-arbutin acted as an inhibitor for the reduction of the enzyme activity in the steady state and there was one characteristic of a lag period during oxidation of tyrosine and the lag time was extended significantly with the increasing concentration of α-arbutin. To reveal these, it should be refered to the suicide inactivation of the active site of tyrosinase by α-arbutin. Tyrosinase undergoes an inactivation process when it reacts with its phenolic substrate, a phenomenon that has long been known in the case of enzymes from a variety of natural sources, including fungi, plants and animals [33]–[35]. The active site of tyrosinase contains two copper atoms and the enzyme can occur in three forms: oxy-tyrosinase, the only form reacts with monophenol; deoxy-tyrosinase, binds dioxygen to form oxy-tyrosinase; met-tyrosinase, cannot bind oxygen but is reduced to deoxy-tyrosinase by catechols. To date, three mechanisms have been proposed to explain this inactivation: (i) an attack by the o-quinone product on a sensitive nucleophilic group near the active site [36], (ii) free radical attack on the active site by the reactive oxygen species generated during the processs of oxidation [35], and (iii) a mechanism that tyrosinase regards the catechol substrate as a cresol at the active site, ‘cresolase presentation’. This leads to the catechol being oxidized to form a product able to undergo deprotonation and reductive elimination, resulting in inactivation of the enzyme through releasing copper(0) at the active site [37]. It was reported that the enzyme obtained after purification, is found to be a mixture of > 85% met and <15% oxy forms [38]. Native tyrosinase occurs mainly as met-tyrosinase, which cannot oxidise phenols, e.g. tyrosine, and needs to be reduced to deoxy-tyrosinase by a catechol before phenol oxidation can begin [39]. This activating catecholic substrate is generated indirectly by fast redox exchange of dopaquinone formed slowly by the small amount of oxy-tyrosine present in native tyrosinase. The lag period ends when all the enzyme has been activated by this indirect and relatively slow non-enzymatic formation of dopa [40]–[41]. A previous work showed that all o-diphenols and triphenols are suicide substrates of tyrosinase, the most potent being pyrogallol [42]. From the chemical structure, arbutin and triphenol have similarities. Moreover, previous study have shown that L-ascorbic acid and D-ascorbic acid (Chemical structure of ascorbic acid is shown in Fig. 6) behave like a suicide substrate when tyrosinase acts on them in aerobic conditions [43]. When the α-arbutin was added to the reaction system in aerobic condition, mushroom tyrosinase was proposed to regard α-arbutin as analogue of triphenol, which was a suicide substrate for the tyrosinase. However, the reaction between oxy-tyrosinase and triphenol does not lead to inactivation of all oxy-tyrosinase, there is a partition ratio between the catalytic and the inactivation pathways [42], [44]. On the one hand, under aerobic conditions, the enzymatic form E_ox_ (oxy-tyrosinase) is responsible for such enzymatic inactivation [44]. On the other hand, tyrosine substrate can only react with oxy-tyrosinase. So, it was proposed that the suicidal inactivation by α-arbutin would cause decreased concentration and activity of the small amount of oxy-tyrosinase present in tyrosinase. Such,with increasing concentration of α-arbutin, it showed inhibition of monophenolase activity of mushroom tyrosinase, and led to an increase in the lag time, meanwhile. Furthermore, the lag time is dependent on various factors such as substrate and enzyme concentration, enzyme source, pH of the medium, presence of a hydrogen donor such as L-dopa or other catechols and transition metal ions [45].

For the diphenolase activity, α-arbutin acted as an activator and there was no lag period during oxidation of L-Dopa. Since both the oxy-tyrosinase and met-tyrosinase can react with catechol(such as L-Dopa), there is no lag time during the diophenolase activity of mushroom tyrosinase. Although the suicidal inactivation of the oxy-tyrosinase will occur for the addition of α-arbutin, there is only a relatively small portion of the oxy-tyrosinase inactivation. Compared with the activation of tyrosinase by α-arbutin, suicide inactivation of oxy-tyrosinase appears to be negligible during the diphenolase activity. But why α-arbutin could activate the mushroom tyrosinase during the oxidation of L-Dopa. It was proposed that α-arbutin induces a conformational change in normal tyrosinase, which not only makes the binding of L-Dopa to the enzyme more effective, but also greatly increases the velocity of its transformation. Monod et al. first presented the word of allosteric to illurstrate the effect of non-substrate-like molecules on enzyme activity, which cause cooperativity between the subunits of an enzyme [46]. Although conformational flexibility of enzymes is reported to be the main point for cooperation, allostery phenomena of many allosteric enzymes are different from each other [47]. As we know, sodium dodecyl sulphate (SDS) is an anionic detergent that inactivates most enzymes. In contrast, tyrosinase and other type 3 copper proteins have been shown to be activated by SDS and it was found to involve changes in the protein tertiary structure [48]. Inaddition previous studies showed that both methionine residues are essential for diphenolase activity [49]. Possibly, by participating in hydrophobic interactions with the aromatic ring of L-dopa, these residues contribute to the “correct” orientation of the substrate in the binding site and decrease the effect of suicide inactivation [50]–[51]. It seems that, the activation of diphenolase activity is due to the interaction of α-arbutin with residues located at the entrance to the active site and the decrease of the effect of suicide inactivation.
